# Sexually Transmitted Infections among Heterosexual Male Clients of Female Sex Workers in China: A Systematic Review and Meta-Analysis

**DOI:** 10.1371/journal.pone.0071394

**Published:** 2013-08-12

**Authors:** Megan M. McLaughlin, Eric P. F. Chow, Cheng Wang, Li-Gang Yang, Bin Yang, Jennifer Z. Huang, Yanjie Wang, Lei Zhang, Joseph D. Tucker

**Affiliations:** 1 UNC Project – China, Guangzhou, China; 2 The Kirby Institute, University of New South Wales, Sydney, Australia; 3 Guangdong Provincial Center for STI & Skin Diseases Control, Guangzhou, China; 4 Department of International Health, School of Nursing and Health Studies, Georgetown University, Washington, District of Columbia, United States of America; Vanderbilt University, United States of America

## Abstract

**Background:**

Female sex workers have been the target of numerous sexually transmitted infection (STI) prevention strategies in China, but their male clients have attracted considerably less public health attention and resources. We sought to systematically assess the prevalence of HIV, syphilis, gonorrhea, and chlamydia among heterosexual male clients of female sex workers in China.

**Methods/Principal Findings:**

Original research manuscripts were identified by searching Chinese and English language databases, and 37 studies analyzing 26,552 male clients were included in the review. Client STI prevalence across studies was heterogeneous. Pooled prevalence estimates and 95% confidence intervals were 0.68% (0.36–1.28%) for HIV, 2.91% (2.17–3.89%) for syphilis, 2.16% (1.46–3.17%) for gonorrhea, and 8.01% (4.94–12.72%) for chlamydia.

**Conclusions/Significance:**

The pooled prevalence estimates of HIV, syphilis, gonorrhea, and chlamydia among clients in this review exceed the prevalences previously reported among population-representative samples and low-risk groups in China. However, heterogeneity across studies and sampling limitations prevent definitive conclusions about how the prevalence of STIs in this population compares to the general population. These findings suggest a need for greater attention to clients’ sexual risk and disease prevalence in China’s STI research agenda in order to inform effective prevention policies.

## Introduction

China has a history of both large epidemics of sexually transmitted infections (STIs) and comprehensive population-based responses. In the 1950s, the Chinese government launched a massive campaign to eliminate STIs through screening, free penicillin, and suppression of the commercial sex industry [1], [2]. Although there were limited epidemiological investigations, by the 1960s STIs were thought to be extremely uncommon [2]. In the wake of extensive economic and social change since the 1980s, both commercial sex and STIs have experienced a marked resurgence in China [3]. The rising prevalence of syphilis and other STIs in the general population in China suggests the potential for the HIV epidemic in China to accelerate through sexual transmission [3]–[9]. Heterosexual transmission has emerged in recent years as the primary mode of HIV transmission in China, accounting for 62.6% of estimated new infections in 2009, compared to 11.3% prior to 2006 [10]–[12].

Subgroups of heterosexual men in China are likely at an increased risk for STIs and HIV infection. One male heterosexual risk group in China that has attracted concern is “mobile men with money”–wealthy businessmen and officials who purchase unprotected sex [13]. Another male heterosexual group believed to be at increased risk of STIs are “surplus men”–an increasing population of young, poor, unmarried men, resulting from China’s imbalanced sex ratio [14]. This group of men may be unable to afford a bride price but able to pay the price of a low-fee sex worker [14]. Understanding these and other subgroups of heterosexual Chinese men who purchase sex is important for designing effective STI/HIV prevention strategies. China’s national median prevalence of commercial sex purchased by men is 4.2% (95% confidence interval [CI], 3.5%–5.2%), exceeding the median prevalence of 2.4% among 55 other countries [15]. The existence of both a significant amount of data on the health and behavior of male clients and population-based data on STI prevalence makes China a uniquely appropriate place to study the STI/HIV risk of clients.

Despite the theoretical STI risk of heterosexual male clients of female sex workers (FSW), there are reasons why clients in China may not have high STI prevalence. Although the higher prevalence of STIs among FSW in China compared with the general population has already been well described [11], [16]–[18], clients often report higher rates of condom use with commercial sex partners compared with wives or other stable partners [10], [19]–[23]. Clients may also seek formal STI care more promptly than men who do not purchase sex [24]–[26]. Thus, research is needed to better understand the sexual risk and prevalence of STIs among clients.

Compared to the literature on FSW, relatively few published studies to date have examined heterosexual male clients of FSW in China [27]. Clients are a heterogeneous and sometimes transient group, making it difficult to sample from the population and produce generalizable findings. Additionally, in a setting where commercial sex work is punished and highly stigmatized [28], clients can be difficult to reach and hesitant to participate in surveys.

We abstracted cross-sectional prevalence data, recruitment methods, and other information on heterosexual men who purchase sex from FSW. The purpose of this study is to systematically assess the prevalence of HIV, syphilis, gonorrhea, and chlamydia among heterosexual male clients of FSW in China.

## Methods

### Search Strategy

Studies investigating the prevalence of STIs among heterosexual male clients of FSW were identified by searching for articles in the PubMed, Embase, China National Knowledge Infrastructure (CNKI), Wanfang Data, and CQVIP databases. CNKI, Wanfang Data, and CQVIP are China’s three major online bibliographic databases for searching Chinese-language biomedical journals. Combinations of terms for China, STIs, sex work, and sex workers were used to screen for potentially relevant studies (see Text S1). Chinese search terms incorporated the variety of local expressions for sex workers reported in the public health and social science literature [29]. Articles from all years were eligible for inclusion. All database searches were updated through December 31, 2012.

Following PRISMA guidelines, the list of publications obtained through this initial search was narrowed to studies relevant to our analysis (see Table S1) [30]. Studies that reported the quantitative prevalence of biomarkers of HIV, syphilis, gonorrhea, or chlamydia among male clients in mainland China or Hong Kong were included. Because the English and Chinese search terms did not limit the initial search to clients, the first round of exclusion based on article titles eliminated a large number of publications on the subject of female sex workers that did not report on heterosexual male clients. Male clients were defined as men who had ever purchased sex from a female sex worker in their lifetime. Although men who sell sex to men are also an important HIV risk group, this review included only studies of heterosexual male clients of FSW (referred to hereafter as “clients”). Reviews, intervention studies, modeling studies, and studies lacking sufficient details on methods were excluded. Studies were included if the sample consisted entirely of clients or if the sample was a more general group of men (e.g., miners or patients at STI clinics) but the reported STI prevalence was disaggregated by whether the men reported having purchased sex.

### Data Extraction and Analysis

For all studies, two study authors (MMM and CW) independently extracted the following data: study location (province and city), population sampled, recruitment methods, total sample size, number of clients in the sample, years of data collection, age, income, marital status, definition of client, biomarkers examined, and prevalence of HIV, syphilis, gonorrhea, and chlamydia. The four STIs were defined according to national laboratory standards for diagnosis [31].

Publication bias was examined by visually inspecting funnel plots for asymmetry; for each STI a plot of the prevalence (x-axis) and the sample size (y-axis) was examined [32]. The Begg and Mazumdar rank correlation was also used to evaluate publication bias (p-value <0.05 represents significant publication bias) [33]. Heterogeneity across studies was estimated by calculating I^2^, an index of the variation across studies that is due to heterogeneity as opposed to chance [34]. The studies were also categorized by population sampled (detainees vs. other), sampling strategy (convenience sample vs. other), region (southwest vs. other), and study period (2000–3, 2004–6, 2007–9, 2010–12), and these categories were examined for differences in the I^2^ statistic. Study locations were categorized based on official domestically used designations [35]. Detainees refer to men arrested for purchasing sex and detained in jail or detention facilities known as reeducation centers, where they may be tested for HIV and other STIs [28].

Random effect models were used to calculate the pooled prevalence estimates and 95% confidence intervals (CI) for HIV, syphilis, gonorrhea, and chlamydia. The pooled prevalence estimates were also calculated by population sampled, sampling strategy, location, and study period, and the chi-squared based Cochrane Q-test was used to compare the pooled prevalence estimates between subgroups. Odds ratios and 95% CI were calculated for the STI prevalence estimates to compare the risk of infection between clients and the general adult population in China. Comprehensive Meta-Analysis Version 2 (Biostat, Englewood, NJ) was used for all analyses.

## Results

**Figure 1:**
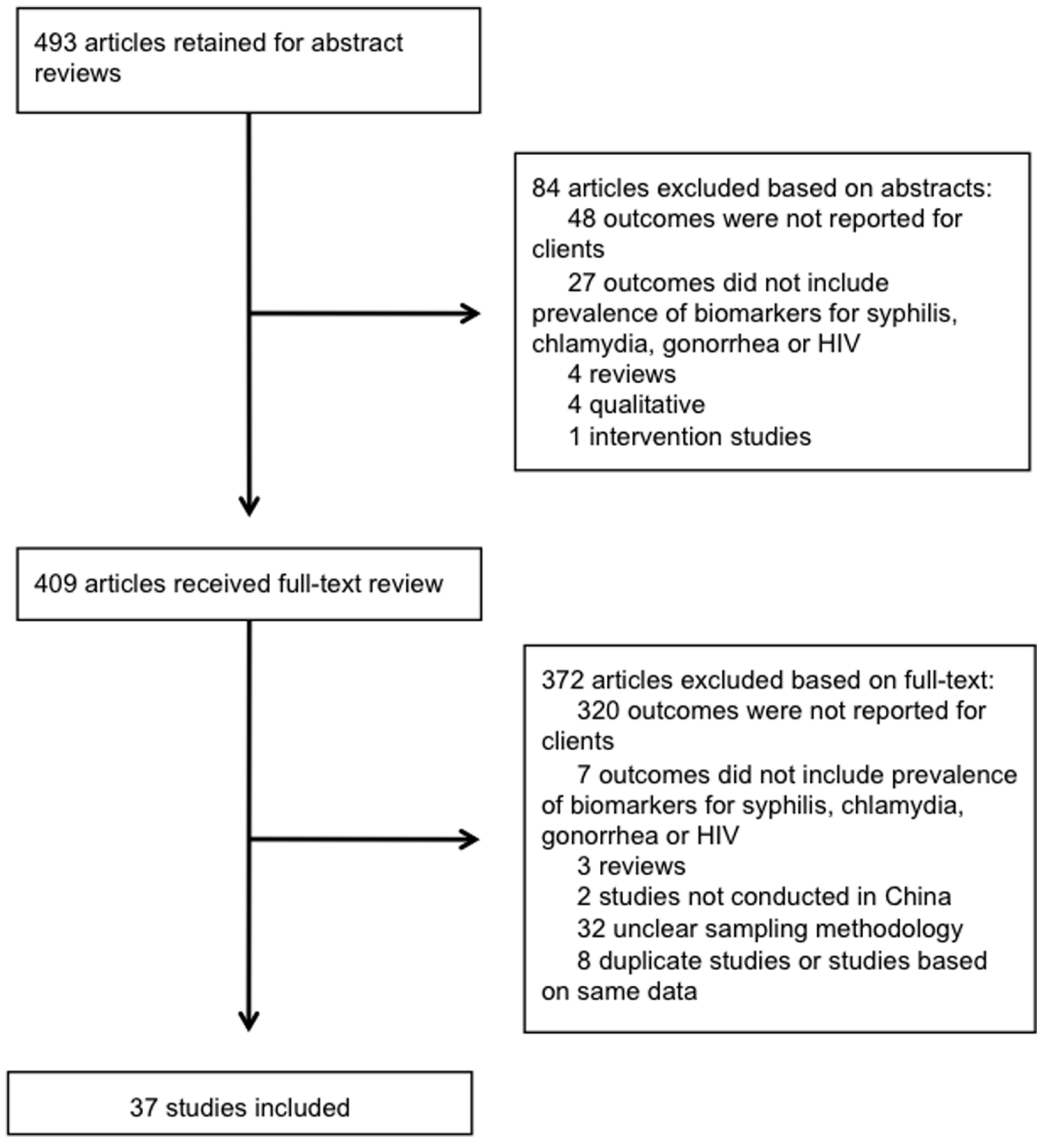
Summary of literature search and study selection.

2376 publications were obtained through the literature search, of which 37 met the inclusion criteria (Figure 1). There were large differences in the prevalence of HIV, syphilis, gonorrhea, and chlamydia reported by the studies (Table 1).

**Table 1:** Characteristics of studies examining STI prevalence among heterosexual male clients of female sex workers in China (N = 37).

First Author	Location (Region)	Years	Population Sampled	Sampling Method	Age	Total Sample Size	Number of Clients	HIV Prevalence	Syphilis Prevalence	Chlamydia Prevalence	Gonorrhea Prevalence
Jia et al. [12]	Kunming, Yunnan (Southwest)	1995–2007	Detainees *	Convenience sample	–	57325	4501	1995–1997∶0.0%; 1998∶0.3%; 1999–2004∶0.3–1.8%; 2005–2007∶0.8–1.8%	–	–	–
Zhang et al. [54]	Nanchong, Sichuan (Southwest)	2004–2005	Detainees	Convenience sample	31% <20 years; 44% 20–29 years; 17% 30–39 years; 8% >40 years **	213	68	0.0%	2.9%	–	–
Zhang et al. [49]	Shangqiu, Henan (South Central)	2000	Detainees	Convenience sample	17.56% 20–30 years; 39.63% 30–40 years; 34.74% 50–60 years; 8.07% 50–60 years	96	31	0.0%	3.2%	–	3.2%
Zhu et al. [50]	Changsha, Hunan (South Central)	2001–2004	Detainees	Convenience sample	–	1634	1634	–	2.1%	–	–
Lu et al. [51]	Zhuhai, Guangdong (South Central)	2003–2007	Detainees	Convenience sample	Mean (range): 21.8 (16–47) years	2546	221	0.0%	3.6%	13.1%	2.3%
Wang et al. [52]	Dongguan, Guangdong (South Central)	2007	Detainees	Convenience sample	Mean (range): 32.97 (19–57) years	358	130	0.0%	9.2%	–	–
Hua et al. [37]	Wuxi, Jiangsu (East)	2001–2005	Detainees	Convenience sample	Mean (SD): 40.96 (11.19) years	832	527	–	1.7%	–	–
Zhu et al. [39]	Shanghai municipality (East)	2002–2005	Detainees	Convenience sample	1.32% <20 years; 20.05% 20–29 years; 26.43% 30–39 years; 34.36% 40–49 years; 17.84% ≥50 years	1011	454	0.0%	–	–	–
Ni et al. [57]	Haiyan County, Zhejiang (East)	2004–2008	Detainees	Convenience sample	Range: 17–73 years	477	477	0.0%	8.0%	5.2%	1.9%
Zhang et al. [36]	Shanghai municipality (East)	2005	Detainees	Convenience sample	4.9% <20 years; 40.0% 20–40 years; 55.1% >40 years	5701	2033	–	2.9%	–	3.4%
Zeng et al. [58]	Shanghai municipality (East)	2005–2007	Detainees	Convenience sample	1.35% ≤20 years; 32.66% 21–30 years; 41.67% 31–40 years; 19.37% 41–50 years; 4.05% 51–60 years; 0.9% ≥61 years	892	444	0%	2.9%	1.4%	2.3%
Xi et al. [59]	Wuxi, Jiangsu (East)	2007–2011	Detainees	Convenience sample	–	931	497	0.0%	2.2%	–	0.2%
Hong et al. [60]	Huzhou, Zhejiang (East)	2008–2009	Detainees	Convenience sample	–	1227	656	0.2%	1.5%	–	0.8%
Qiu et al. [61]	Hangzhou, Zhejiang (East)	2008–2010	Detainees	Convenience sample	Mean (range): 36 (16–71) years	423	423	0.2%	7.8%	9.5%	0.5%
Wu et al. [62]	Shanghai municipality (East)	2008–2011	Detainees	Convenience sample	4.30% <21 years; 35.56% 21–30 years; 34.17% 31–40 years; 18.33% 41–50 years; 7.64% >50 years	1142	720	–	4.9%	–	3.5%
Chen et al. [63]	Zhuji, Zhejiang (East)	2009–2011	Detainees	Convenience sample	32.08% 20–29 years; 27.2% 30–39 years	1172	592	0.0%	2.4%	–	1.9%
Hu et al. [64]	Beijing municipality (North)	1999–2000	Detainees	Convenience sample	Mean (SD): 31.19 (0.41); range: 17–67 years	1086	566	0.0%	0.9%	–	8.3%
Zhao et al. [65]	Beijing municipality (North)	2001–2002	Detainees	Convenience sample	Maximum: 66 years	400	400	0.0%	1.3%	22.0%	1.8%
Li et al. [66]	Beijing municipality (North)	2001–2004	Detainees	Convenience sample	2001∶58.76% 20–29 years; 2002∶40.09% 30–39 years; 2003∶41.45% 30–39 years; 2004∶42.76% 30–39 years	2517	1005	0.1%	1.7%	7.1%	2.1%
Liu et al. [67]	Beijing municipality (North)	2005	Detainees	Convenience sample	–	512	53	–	1.9%	–	–
Li et al. [68]	Beijing municipality (North)	2005–2008	Detainees	Convenience sample	–	2888	2888	0.1%	2.4%	–	–
Gao et al. [70]	Beijing municipality (North)	2006	Detainees	Convenience sample	Range: 15–53 years	311	32	0.0%	0.0%	–	–
Dong et al. [69]	Tianjin municipality (North)	2007	Detainees	Convenience sample	Median (range): 36 (18–57) years	186	186	0.0%	1.6%	–	–
Liu et al. [41]	Liuzhou, Anhui (East)	2012	Detainees, commercial sex venue attendees, and STI clinics attendees	Convenience sample	Mean (SD): 49.97 (8.78) years; range: 39–81 years; 87.25% 39–60 years	400	400	2.3%	8.0%	–	–
Zhang et al. [46]	Gejiu, Yunnan (Southwest)	2006	Miners at five selected mines	Convenience sample	Mean (SD): 27.5 (6.7) years; range: 16–52 years	1798	336	1.5%	2.4%	6.9%	2.1%
Yao et al. [56]	Yunnan province (Southwest)	2007	Commercial sex venue attendees	Convenience sample	22.2% <30 years; 44.4% 30–39 years; 33.3% ≥40 years	36	36	5.6%	–	–	–
Jin et al. [42]	Kaiyuan, Yunnan (Southwest)	2008	FSW clients	Snowball sampling using FSW-client and client-client referrals	Median (IQR): 36 (23–45) years	315	315	6.0%	–	–	–
Ma et al. [43]	Kaiyuan, Yunnan (Southwest)	2008	Commercial sex venue attendees	Convenience sample	Mean (SD): 33.94 (12.56) years; range: 16–75 years	775	558	4.7%	2.3%	–	–
Lei et al. [55]	Chongqing municipality (Southwest)	2008	STI clinic attendees	Convenience sample	Median (range): 35 (17–83) years	514	514	1.4%	12.3%	–	–
Yang et al. [47]	Xichang/Zigong/Leshan, Sichuan (Southwest)	2008	FSW clients	Snowball sampling using referrals from clients identified during VCT, at methadone clinics, and at commercial sex venues	Median (range): 38 (18–75) years	600	600	1.5%	5.3%	–	–
Feng et al. [38]	Sichuan province (Southwest)	2008–2009	FSW clients and VCT clinic attendees	Convenience sample	Median: 34 years (2008), 44 years (2009)	2386	2386	0.2%	1.7%	–	–
Zhang et al. [44]	Yunnan province (Southwest)	2009	FSW clients	Convenience sample	5% 16–25 years; 23% 26–35 years; 47% 36–45 years; 25% >45 years	154	57	29.8%	–	–	–
Reilly et al. [48]	Hekou county, Yunnan province (Southwest)	2010	Commercial sex venue attendees	Snowball sampling through FSWs and their bosses, and other clients	Median (range): 36 (18–70) years	306	306	9.2%	0.0%	–	–
Yang et al. [53]	Yichang, Hubei (South Central)	2008–2010	STI clinic attendees	Convenience sample	Median (range): 32.43 (18–83) years	450	450	0.4%	4.6%	–	–
Liu et al. [45]	Unnamed city in Guangxi province (South Central)	2010	Commercial sex venue attendees	Convenience sample	Median: 56 years; 65.62% >50 years; 10.42% >75 years	148	108	0.0%	8.4%	–	–
Parish et al. [4]	National	1999–2000	General population	Stratified population probability sampling	15% 20–24 years; 50% 25–34 years; 35% 35–44 years **	3426	106	–	–	10.4%	–
Wang et al. [71]	Five unnamed cities	2009	Commercial sex venue and STI clinic attendees	Convenience sample	90% 20–60 years	1842	1842	0.1%	1.6%	–	–

**NOTE:** STI = sexually transmitted infection; IQR = interquartile range; VCT = voluntary counseling and testing; SD = standard deviation; FSW = female sex worker.

* Males detained at reeducation centers or jails for purchasing sex, or men arrested and sent by the public security bureau for STI testing.

** Among the total sample.

The median number of clients included in the studies was 450 (interquartile range [IQR], 186–2033). The total sample size of clients for all 37 studies was 26,552. Among studies reporting median age or age distributions, the majority of clients sampled were between 25 years old and 45 years old, except for six studies reporting a higher proportion of clients over 45 years of age [36]–[41]. Among studies reporting marital status, the majority of clients were married or living with their regular partner; the proportion exceeded 50% in all studies except four [42]–[45] and exceeded 75% in eight studies. Only six studies reported the income levels of the clients sampled. Three studies reported a median annual income between 1300–2000 US$ [42], [45], [46]. In the other three studies, 40% of participants earned less than 1700 US$ per year [47], 65% earned less than 2900 US$ per year [43], and 33% earned less than 800 US$ per year [48].

Six studies were undertaken in south central China [45], [49]–[53], eleven in the southwest [12], [38], [42], [44], [46]–[48], [53]–[56], eleven in the east [36], [37], [39], [41], [57]–[63], and seven in the north [64]–[70]. Two studies were national [4] or multi-city samples [71]. There were no studies from northwestern or northeastern regions in China. Most studies used convenience sampling, but three studies used snowball sampling with female sex worker or client-client referrals [42], [47], [48], and one used stratified probability sampling [4]. Twenty-four studies sampled detainees [12], [36], [37], [39], [41], [49]–[52], [54], [57]–[70], but other studies sampled miners [46], clients at commercial sex venues [38], [41]–[43], [45], [47], [48], [56], [71], STI clinic attendees [38], [41], [47], [53], [55], [71], or the general population [4]. Four studies defined clients as men who reported purchasing sex in the previous year [4], [47], [48], [55]. Other studies defined clients as men who reported purchasing sex in the previous three months [43] or “recently” [71]. Two studies included only men who sought sex at a commercial sex venue at the time they were recruited [45], [56]. The remaining studies defined clients as men who reported ever purchasing sex [44], [46], [53] or did not specify the time period when sex was purchased.

The Begg and Mazumdar rank correlation test indicated that there was no publication bias among the studies reporting HIV (p = 0.353), syphilis (p = 0.331), gonorrhea (p = 0.139), or chlamydia (p = 0.322) prevalence. Visual inspection of the funnel plots also revealed no significant publication bias among the studies. There was no recognizable association observed between study size and reported prevalence. The prevalence of each STI was heterogeneous between studies, with an I^2^ statistic greater than 80 for gonorrhea and greater than 90 for HIV, syphilis, and chlamydia.

There was no significant change in the I^2^ statistic when the studies reporting syphilis prevalence were subdivided by study location, population sampled, or sampling method (Table 2). Among the studies reporting HIV prevalence, studies in southwest China showed greater heterogeneity than those undertaken in other regions (95.8 vs. 62.9), and studies of detainees showed less heterogeneity than those studying other populations of clients (60.3 vs. 94.2). There was no significant change in the I^2^ statistic when the studies reporting HIV prevalence were subdivided by sampling method (Table 3). For both HIV and syphilis, studies conducted between 2007 and 2012 had high heterogeneity (>65) whereas those conducted prior to 2007 had no significant heterogeneity. Forest plots of the prevalence of HIV, syphilis, gonorrhea, and chlamydia among clients of FSW are presented in the (see Figures S1 to S4 in File S1).

**Table 2:** Subgroup analyses for HIV prevalence among male clients of female sex workers in China.

Characteristic	Subgroup	Number of studies	Pooled prevalence, % (95% CI)	p-value *	I-square
Overall	–	31	0.68 (0.36–1.28)	–	92.20
Study location	Southwest	11	2.54 (1.07–5.91)	<0.01	95.83
	Other	20	0.24 (0.12–0.49)		62.89
Population sampled	Detainees	19	0.30 (0.16–0.56)	<0.01	60.33
	Other	12	2.00 (0.88–4.50)		94.23
Sampling method	Convenience sampling	28	0.50 (0.24–1.03)	<0.01	90.84
	Other	3	4.56 (1.77–11.23)		91.54
Study period	2000–2003	6	0.23 (0.07–0.70)	0.812	0
	2004–2006	8	0.30 (0.11–0.80)		0
	2007–2009	18	0.39 (0.12–1.25)		89.92
	2010–2012	4	0.81 (0.06–10.62)		85.42

**NOTE:** CI = confidence interval.

* P-value from chi-squared based Cochran Q-test was used to compare the pooled prevalence estimates between subgroups, *p*<0.05 indicates significant difference.

**Table 3:** Subgroup analyses for syphilis prevalence among male clients of female sex workers in China.

Characteristic	Subgroup	Number of studies	Pooled prevalence, % (95% CI)	p-value *	I-square
Overall	–	31	2.91 (2.17–3.89)	–	90.94
Study location	Southwest	7	2.13 (0.80–5.51)	0.465	94.97
	Other	24	3.09 (2.34–4.06)		87.17
Population sampled	Detainees	22	2.98 (2.23–3.98)	0.766	86.22
	Other	9	2.65 (1.26–5.47)		95.16
Sampling method	Convenience sampling	29	2.98 (2.23–3.98)	0.622	91.31
	Other	2	2.65 (1.26–5.47)		83.71
Study period	2000–2003	9	0.28 (0.11–0.70)	<0.01	0
	2004–2006	12	0.30 (0.14–0.67)		0
	2007–2009	15	1.13 (0.54–2.34)		87.60
	2010–2012	4	8.79 (5.36–14.07)		65.99

**NOTE:** CI = confidence interval.

* P-value from chi-squared based Cochran Q-test was used to compare the pooled prevalence estimates between subgroups, *p*<0.05 indicates significant difference.

Among the 31 studies reporting HIV prevalence, the pooled estimate of HIV prevalence among clients was 0.68% (95% CI 0.36–1.28%) (Table 2). The HIV prevalence estimate among southwestern studies was higher than the HIV prevalence estimate among studies undertaken in other regions (2.54% vs. 0.24% p = 0.001). The estimated HIV prevalence among studies that used convenience sampling was significantly lower compared to those that used other sampling methodologies (0.50% vs. 4.56%, p<0.01). The estimated prevalence among studies that sampled detainees was lower than among studies that sampled other subgroups of clients (miners, STI clinic attendees, men at commercial sex work venues, or the general population) (0.30% vs. 2.00%, p<0.01). Among studies reporting data for one or two year intervals, there was a non-significant increase in HIV prevalence in later years (0.23% in 2000–3, 0.30% in 2004–6, 0.39% in 2007–9, 0.81% in 2010–12, p = 0.812). Clients were found to have 11.80 (95% CI 10.03–13.88) times greater odds of being infected with HIV than the general Chinese population (Table 4).

**Table 4:** Odds ratios and 95% confidence intervals for the prevalence of STIs among male clients of FSW compared to the general population in China.

Disease prevalence	Adults in general population			Male clients of FSW		OR (95% CI)
	Total sample size	Estimated prevalence, % (95% CI)	Source	Total sample size	Pooled prevalence estimate, % (95% CI)	
HIV	1,344,130,000	0.06 (0.05–0.07)	[11]	21,479	0.68 (0.36–1.28)	11.80 (10.03–13.88)*
Syphilis	17,226	0.52 (0.33–0.71)	[72]	21,083	2.91 (2.17–3.89)	5.73 (4.59–7.16)*
Gonorrhea	3,426	0.05 (0.01–0.22)	[4]	8,401	2.16 (1.46–3.17)	44.10 (9.79–198.64)*
Chlamydia	3,341	3.29 (2.74–3.95)	[4], [92]	3,412	8.01 (4.94–12.72)	2.56 (2.04–3.21)*

**Note:** OR = odds ratio, CI = confidence interval.

* Indicates the OR is statistically greater than the reference category (general population) at *p*<0.05.

Among the 31 studies reporting syphilis prevalence, the pooled estimate of syphilis prevalence among clients was 2.91% (95% CI 2.17–3.89%) (Table 3). The syphilis prevalence estimate did not differ significantly by region, sampling methodology, or population sampled. Among studies reporting data for one or two year intervals, there was a significant increase in syphilis prevalence in later years (0.28% in 2000–3, 0.30% in 2004–6, 1.13% in 2007–9, 8.79% in 2010–12, p<0.01). Clients were found to have 5.73 (95% CI 4.59–7.16) times higher odds of having syphilis infection compared to the general Chinese population (Table 4).

Among the 14 studies reporting gonorrhea prevalence, the pooled estimate of gonorrhea prevalence among clients was 2.16% (95% CI 1.46–3.17%). Among the eight studies reporting chlamydia prevalence, the pooled estimate of chlamydia prevalence was 8.01% (95% CI 4.94–12.72%). Subgroup analyses were not performed for gonorrhea and chlamydia because the number of studies was insufficient. Compared to the general Chinese population, clients were found to have 44.10 (95% CI 9.79–198.64) and 2.56 (95% CI 2.04–3.21) times greater odds of being infected with gonorrhea and chlamydia, respectively.

## Discussion

While there have been a number of studies examining subsets of high-risk men, there have been few studies that systematically investigate clients of FSW. To our knowledge, this is the first systematic review of STI prevalence among heterosexual male clients of FSW in China. Our review revealed considerable variation in STI prevalence reported among clients in China, as well as substantial variation in sampling strategies used.

The pooled estimates of HIV, syphilis, gonorrhea, and chlamydia prevalence among male clients of FSW in this review exceed the prevalences previously reported in population-representative samples, large samples of low-risk groups, and national estimates in China. The prevalence of HIV in China is currently estimated to be 0.06%, corresponding to 11.8 times lower odds of HIV infection than clients in this review [11]. A large study of couples receiving compulsory premarital examinations found a low prevalence of syphilis across various study sites in China (0.33% to 0.71%) [72]. In comparison, clients in this review were found to have 5.7 times higher odds of being infected with syphilis. In their national stratified probability sample of Chinese individuals ages 20 to 64 years, Parish and colleagues found that among men, the prevalence of gonorrhea was 0.02% (95% CI 0.005–0.1%) and the prevalence of chlamydial infection was 2.1% (95% CI 1.3–3.3%) [48]. Clients in this review had 44.1 times and 2.7 times higher odds of being infected with gonorrhea and chlamydia, respectively.

The prevalence of STIs among clients in this review is lower than the prevalence among other at-risk groups in China reported in systematic reviews. The prevalence of HIV among FSWs in China is estimated to be 0.6%–3.0% [73], [74], compared to 0.68% among clients in our review. And the prevalence of syphilis, gonorrhea, and chlamydia among Chinese FSWs is estimated to be 5.0–6.9% [74], [75], 16.4% [74], and 25.7% [74], respectively, compared to 2.91%, 2.16%, and 8.01% among clients in our review. Among Chinese men who have sex with men, HIV prevalence is estimated to be 5.3% [76] and syphilis, 13.5% [77], compared to 0.68% and 2.91% among clients in our review. However, heterogeneity across studies and sampling limitations prevent definitive conclusions about how the prevalence of STIs in this population compares to the general population and other at-risk groups.

Most of the studies included in this systematic review used a convenience sample of detainees at jails or reeducation centers. The prevalence of HIV was lower among detainees compared to other populations. This trend is the opposite of what has been found among FSW in China. Systematic reviews have shown that incarcerated FSW have a higher prevalence of syphilis compared to FSW who have been sampled at entertainment centers, beauty salons, and other places of work [5], [28]. Moreover, the trend toward lower HIV infection among clients who have been detained is surprising given the literature on incarceration as a risk factor for STIs [78]–[85]. However, male client detainees in China differ from typical incarcerated populations in a crucial way–they are detained specifically for purchasing sex. In other contexts where prisoners have been detained for a variety of offenses, the reason for incarceration may affect the association between incarceration and STIs. Income and other variables may confound these relationships, but the high degree of heterogeneity among the studies in terms of both STI prevalence and reported demographic characteristics precluded a meta-analysis of income or other predictors. Additionally, a number of the studies that did not examine detainees sampled clients seeking care at STI clinics, a population that would be expected to have a higher prevalence of STIs, and this may in part explain the difference in HIV prevalence found between detainees and other subgroups.

Accurately sampling clients is challenging. Male clients of FSW comprise a heterogeneous group, representing men of varying income, education, and occupational categories [15], [86]. Men who frequently purchase commercial sex may be oversampled in studies of clients, which could lead to an overestimation of STI prevalence in the male client population. Stigmatization of commercial sex work and detention of clients during crackdown campaigns further complicates sampling and outreach programs. Similar problems identifying clients and enrolling them in research studies have been reported in other contexts [87], [88], [89]. Moreover, the definition of client that Chinese police have used for arrests has evolved over time, so the meaning of detainee is likely to be somewhat inconsistent across different time periods.

The heterogeneity in the populations sampled and the prevalence of STIs reported across the studies reviewed represents a limitation of the present review and underscores the challenge of sampling clients. This variation across studies also highlights a limitation of systematic review research, as the findings can vary based on the databases and search terms selected. The high degree of heterogeneity also limited our ability to evaluate publication bias, and it is possible that publication bias may have been unidentified. Another limitation was the inclusion of a number of studies of men detained for purchasing sex. Although according to national policies detainees give informed consent for routine health exams that include STI/HIV testing [90], many of the studies did not provide sufficient detail to allow us to evaluate the consent process. Our comparison of the prevalence of STIs among clients versus the general population is limited because a number of client studies sampled men attending STI clinics, who would be expected to have a higher risk of STI infection. Additionally, the data on chlamydia and gonorrhea prevalence in the general population is more than ten years old, so we assumed that these two epidemics have been stable in recent years. Finally, differences among the studies in terms of the definition of male client also limit the findings of this review. Most studies defined a male client as a man who reported ever purchasing sex, and some studies did not specify the definition of client. Future studies examining the prevalence of STIs among clients should consider reporting STI prevalence by frequency of purchasing sex and type of sex worker or commercial sex venue frequented.

This study highlights the need for further community-based sampling strategies among male clients. Differences in the reported prevalence of HIV and syphilis by sampling strategy and population sampled suggest the limitations of convenience sampling. In comparison, population-based probability sampling, used by Parish and colleagues [4], is a more rigorous sampling methodology. Despite its limitations for sampling purposes, detention offers an opportunity for intervention for this hard-to-reach population. Every country will have its own unique policy, but in China, where a large number of men are detained for purchasing sex, detention is an occasion when clients could receive routine STI screening and services.

Much of the HIV and STI prevention agenda has focused on women and men who have sex with men, both in China and worldwide. Heterosexual men–including male clients of FSW– are often left out of this agenda. Whereas female sex workers have been the target of numerous STI prevention strategies in China [91], their clients have attracted considerably less public health attention and prevention resources. The higher pooled estimates of HIV, syphilis, gonorrhea, and chlamydia prevalence among clients in this review, compared to the prevalence estimates previously reported for the general population in China, suggest that greater attention to the sexual risk of male clients of female sex workers may be needed. However, the prevalence of STIs in this population is poorly appreciated using conventional study designs, and additional research is needed to characterize their sexual risk and STI prevalence.

## Supporting Information

Table S1 **PRISMA checklist for systematic reviews and meta-analyses.**
(PDF)

Text S1 **English and Chinese search terms used for the systematic review.**
(PDF)

File S1 **Forest plots for HIV, syphilis, gonorrhea, and chlamydia.** Forest plots showing unadjusted estimates (squares) with 95% confidence intervals (bars) for HIV (Figure S1), syphilis (Figure S2), gonorrhea (Figure S3), and chlamydia (Figure S4) among male clients of female sex workers in China. The pooled prevalence estimate is represented as a red diamond.
(PDF)
