# Supplementary material for: Sexually Transmitted Infections among Heterosexual Male Clients of Female Sex Workers in China: A Systematic Review and Meta-Analysis
Source: PLoS One. 2013 Aug 12;8(8):e71394. doi: 10.1371/journal.pone.0071394 (PMC3741140; doi:10.1371/journal.pone.0071394)
Supplement: Text S1 — English and Chinese search terms used for the systematic review. (PDF) [file pone.0071394.s002.pdf]

Text S1

English search terms:

china[mesh] AND "china"[tw]

prostitution[mesh] OR "sex work"[tiab] OR "sex worker"[tiab] OR "sex workers"[tiab] OR "sex workers"[tiab] OR "prostitution"[tiab] OR "prostitutes"[tiab] OR "prostitute"[tiab] OR “commercial sex"[tiab]

Sexually Transmitted Diseases"[Mesh] OR "STD"[tiab] OR "STDs"[tiab] OR "STI"[tiab] OR "STIs"[tiab] OR "sexually transmitted infection"[tiab] OR "sexually transmitted infections"[tiab] OR "sexually transmitted diseases"[tiab] OR "sexually transmitted disease"[tiab] OR "venereal disease"[tiab] OR "venereal diseases"[tiab] OR aids[sb] OR "chlamydia"[tiab] OR "gonorrhea"[tiab] OR "syphilis"[tiab] OR "hepatitis"[tiab] OR "herpes"[tiab] OR "HPV"[tiab] OR "human papillomavirus"[tiab] OR "genital warts"[tiab] OR "chancroid"[tiab] OR "trichomoniasis"[tiab]

Chinese search terms:

China or Hong Kong: 中国 , 香港

Terms for sex worker: 妓女, 娼妓, 小姐, 三陪小姐, 性工作,性工作者, 性交易, 卖淫, 应召女郎, 站街的, 站街女, 叮咚小姐, 发廊妹 , 发廊小姐, 按摩女, 街女, 二奶, 工棚女, KTV小姐, 應召女郎, 劳动教养所, 娼, 妓, 暗娼, 女性性工作者

Terms for STIs: 性病, 脏病, 性传播感染, 花柳病, 艾滋病毒, 艾滋病, 艾滋, 衣原体, 衣原菌, 淋病, 梅毒, 疱疹, 生殖器疣, 软下疳, 滴虫, 性传播疾病
