# Supplementary material for: Sexually Transmitted Infections among Heterosexual Male Clients of Female Sex Workers in China: A Systematic Review and Meta-Analysis
Source: PLoS One. 2013 Aug 12;8(8):e71394. doi: 10.1371/journal.pone.0071394 (PMC3741140; doi:10.1371/journal.pone.0071394)

Figure S1. Prevalence of HIV in male clients of female sex workers in China. Forest plot showing unadjusted estimates (squares) with 95% confidence intervals (bars). Pooled prevalence estimate is represented as red diamond in this plot.

Study Name	Event rate	Lower limit	Upper limit	n/N	Prevalence and 95% CI	Weight (%) (random)
Chen Z, 2012	0.0008	0.0001	0.0133	0 / 592		2.41
Hu XM, 2002	0.0009	0.0001	0.0139	0 / 566		2.41
Li DL, 2006	0.0010	0.0001	0.0070	1 / 1005		3.12
Li GY, 2010	0.0010	0.0003	0.0032	3 / 2888		3.85
Wang L, 2010	0.0010	0.0002	0.0042	2 / 1842		3.60
Xi XW, 2012	0.0010	0.0001	0.0158	0 / 497		2.41
Ni CM, 2011	0.0010	0.0001	0.0165	0 / 477		2.41
Zhu LD, 2007	0.0011	0.0001	0.0173	0 / 454		2.41
Zeng YB, 2009	0.0011	0.0001	0.0177	0 / 444		2.41
Zhao YR, 2003	0.0012	0.0001	0.0196	0 / 400		2.41
Hong QT, 2010	0.0020	0.0004	0.0110	1 / 656		3.35
Qiu XM, 2010	0.0020	0.0002	0.0166	1 / 423		2.96
Feng L, 2010	0.0020	0.0008	0.0049	5 / 2386		4.05
Lu JC, 2008	0.0023	0.0001	0.0349	0 / 221		2.41
Dong XY, 2009	0.0027	0.0002	0.0412	0 / 186		2.41
Wang XX, 2008	0.0038	0.0002	0.0580	0 / 130		2.41
Yang YL, 2011	0.0040	0.0009	0.0171	2 / 450		3.58
Liu Q, 2012	0.0046	0.0003	0.0690	0 / 108		2.41
Jia M, 2010	0.0070	0.0049	0.0099	32 / 4501		4.35
Zhang ZH, 2006	0.0072	0.0005	0.1055	0 / 68		2.40
Lei SC, 2011	0.0140	0.0068	0.0288	7 / 514		4.16
Zhang G, 2010	0.0150	0.0063	0.0354	5 / 336		4.06
Yang C, 2010	0.0150	0.0078	0.0286	9 / 600		4.21
Gao JM, 2008	0.0152	0.0009	0.2008	0 / 32		2.39
Zhang CJ, 2001	0.0156	0.0010	0.2060	0 / 31		2.39
Liu J, 2012	0.0230	0.0121	0.0433	9 / 400		4.21
Ma WJ, 2010	0.0470	0.0322	0.0680	26 / 558		4.33
Yao Y, 2008	0.0560	0.0141	0.1972	2 / 36		3.62
Jin X, 2010	0.0600	0.0385	0.0922	19 / 315		4.30
Reilly KH, 2012	0.0920	0.0643	0.1299	28 / 306		4.33
Zhang H, 2010	0.2980	0.1940	0.4282	17 / 57		4.26
**Total (95% CI)**	0.0068	0.0036	0.0128			

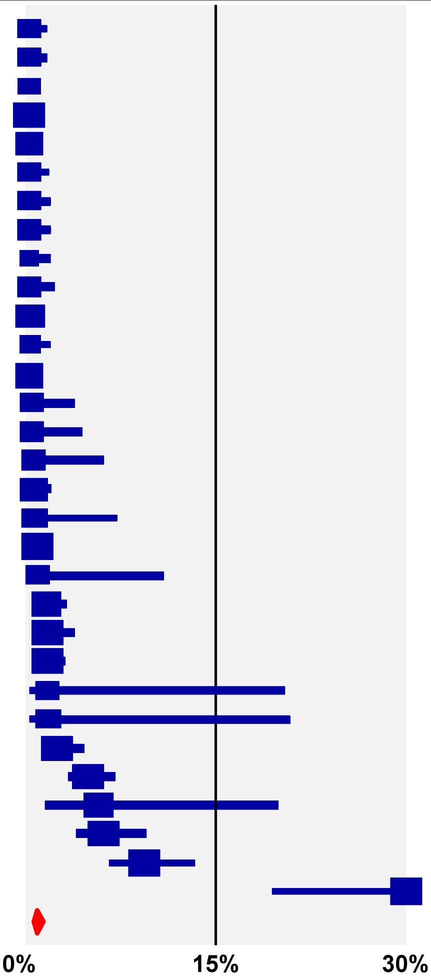

Figure S2. Prevalence of syphilis in male clients of female sex workers in China. Forest plot showing unadjusted estimates (squares) with 95% confidence intervals (bars). Pooled prevalence estimate is represented as red diamond in this plot.

Study Name	Event rate	Lower limit	Upper limit	n/N	Weight (%) (random)
Reilly KH, 2012	0.0016	0.0001	0.0255	0 / 306	0.91
Feng L, 2010	0.0020	0.0008	0.0049	5 / 2386	3.00
Hu XM, 2002	0.0090	0.0038	0.0213	5 / 566	3.04
Zhao YR, 2003	0.0130	0.0055	0.0303	5 / 400	3.06
Hong QT, 2010	0.0150	0.0080	0.0278	10 / 656	3.47
Gao JM, 2008	0.0152	0.0009	0.2008	0 / 32	0.90
Dong XY, 2009	0.0160	0.0051	0.0486	3 / 186	2.56
Wang L, 2010	0.0160	0.0112	0.0229	29 / 1842	3.87
Li DL, 2006	0.0170	0.0106	0.0271	17 / 1005	3.71
Hua YX, 2006	0.0170	0.0089	0.0324	9 / 527	3.42
Liu YQ, 2007	0.0190	0.0027	0.1222	1 / 53	1.48
Zhu JC, 2006	0.0210	0.0151	0.0292	34 / 1634	3.90
Xi XW, 2012	0.0220	0.0122	0.0394	11 / 497	3.52
Ma WJ, 2010	0.0230	0.0134	0.0393	13 / 558	3.60
Chen Z, 2012	0.0240	0.0143	0.0400	14 / 592	3.64
Li GY, 2010	0.0240	0.0190	0.0303	69 / 2888	4.00
Zhang G, 2010	0.0240	0.0121	0.0471	8 / 336	3.35
Zhang Y, 2007	0.0290	0.0225	0.0373	59 / 2033	3.98
Zeng YB, 2009	0.0290	0.0169	0.0494	13 / 444	3.60
Zhang ZH, 2006	0.0290	0.0072	0.1096	2 / 68	2.14
Zhang CJ, 2001	0.0320	0.0045	0.1963	1 / 31	1.45
Lu JC, 2008	0.0360	0.0181	0.0704	8 / 221	3.34
Yang YL, 2011	0.0460	0.0301	0.0697	21 / 450	3.77
Wu RH, 2012	0.0490	0.0354	0.0674	35 / 720	3.90
Yang C, 2010	0.0530	0.0377	0.0741	32 / 600	3.87
Qiu XM, 2010	0.0780	0.0560	0.1077	33 / 423	3.88
Ni CM, 2011	0.0800	0.0588	0.1080	38 / 477	3.91
Liu J, 2012	0.0800	0.0571	0.1109	32 / 400	3.87
Liu Q, 2012	0.0840	0.0444	0.1533	9 / 108	3.39
Wang XX, 2008	0.0920	0.0529	0.1552	12 / 130	3.53
Lei SC, 2011	0.1230	0.0973	0.1543	63 / 514	3.97
	0.0291	0.0217	0.0389		

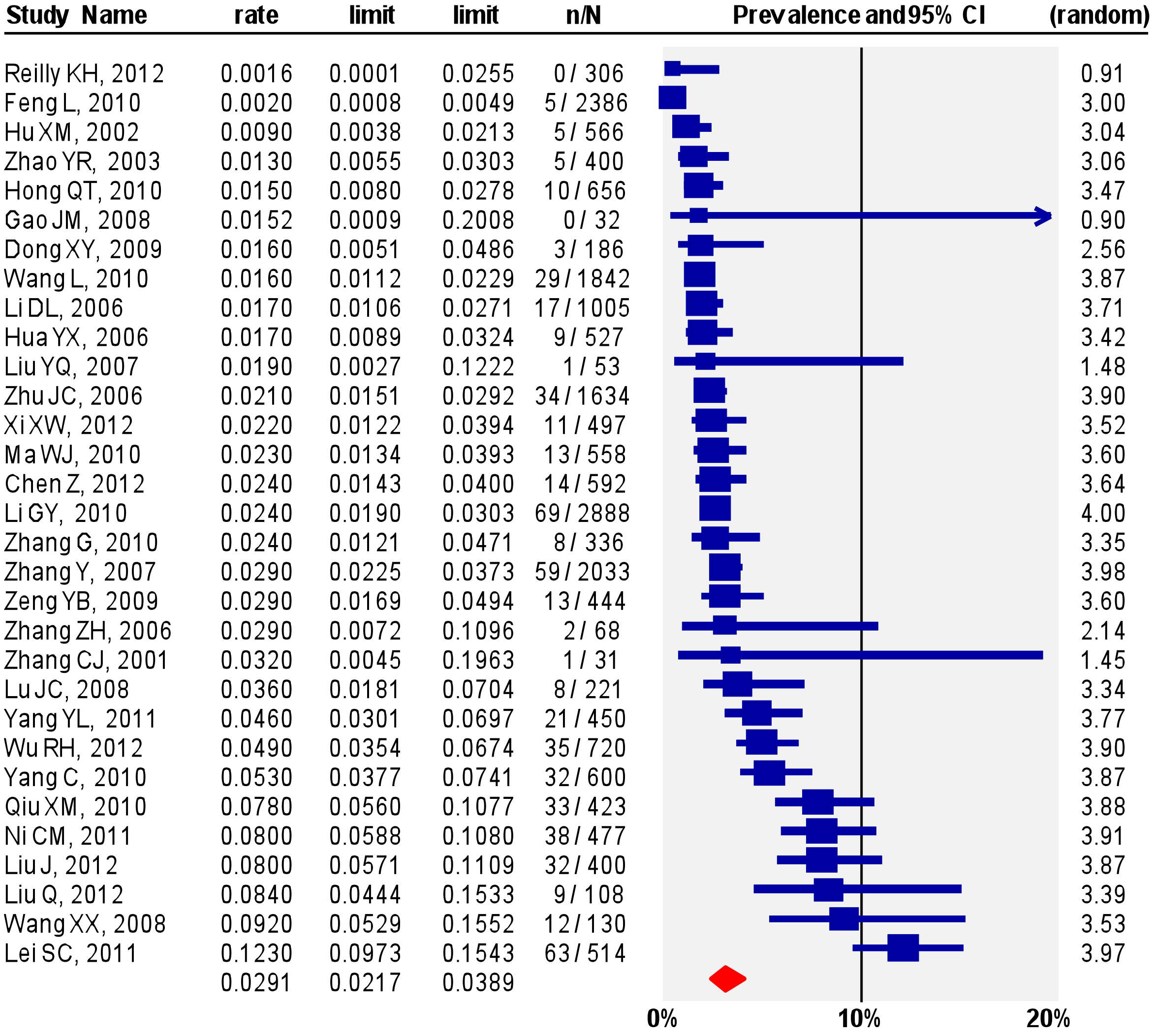

Figure S3. Prevalence of gonorrhea in male clients of female sex workers in China. Forest plot showing unadjusted estimates (squares) with 95% confidence intervals (bars). Pooled prevalence estimate is represented as red diamond in this plot.

Study Name	Event rate	Lower limit	Upper limit	n/N	Weight (%) (random)
Xi XW, 2012	0.0020	0.0003	0.0141	1 / 497	2.88
Qiu XM, 2010	0.0050	0.0013	0.0190	2 / 423	4.63
Hong QT, 2010	0.0080	0.0034	0.0187	5 / 656	6.81
Zhao YR, 2003	0.0180	0.0087	0.0369	7 / 400	7.44
Ni CM, 2011	0.0190	0.0099	0.0360	9 / 477	7.85
Chen Z, 2012	0.0190	0.0106	0.0338	11 / 592	8.20
Li DL, 2006	0.0210	0.0137	0.0320	21 / 1005	8.96
Zhang G, 2010	0.0210	0.0101	0.0433	7 / 336	7.40
Lu JC, 2008	0.0230	0.0097	0.0537	5 / 221	6.71
Zeng YB, 2009	0.0230	0.0125	0.0419	10 / 444	8.04
Zhang CJ, 2001	0.0320	0.0045	0.1963	1 / 31	2.82
Zhang Y, 2007	0.0340	0.0269	0.0428	69 / 2033	9.67
Wu RH, 2012	0.0350	0.0238	0.0512	25 / 720	9.11
Hu XM, 2002	0.0830	0.0629	0.1087	47 / 566	9.49
Total (95% CI)	0.0216	0.0146	0.0317		

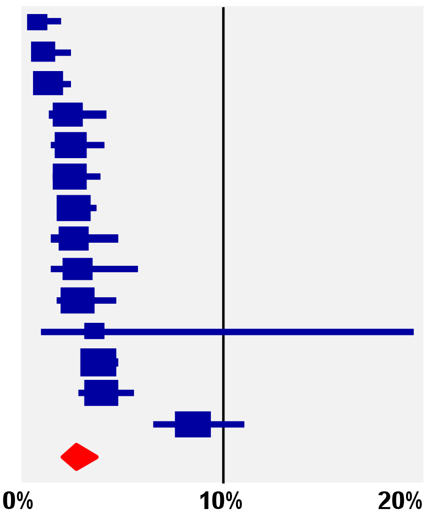

Figure S4. Prevalence of chlamydia in male clients of female sex workers in China. Forest plot showing unadjusted estimates (squares) with 95% confidence intervals (bars). Pooled prevalence estimate is represented as red diamond in this plot.

Study Name	Event rate	Lower limit	Upper limit	n/N	Weight (%) (random)
Zeng YB, 2009	0.0140	0.0064	0.0304	6 / 444	10.42
Ni CM, 2011	0.0520	0.0353	0.0759	25 / 477	12.73
Zhang G, 2010	0.0690	0.0464	0.1015	23 / 336	12.64
Li DL, 2006	0.0710	0.0567	0.0886	71 / 1005	13.41
Qiu XM, 2010	0.0950	0.0705	0.1269	40 / 423	13.09
Parish WL, 2003	0.1040	0.0586	0.1780	11 / 106	11.49
Lu JC, 2008	0.1310	0.0925	0.1822	29 / 221	12.80
Zhao YR, 2003	0.2200	0.1821	0.2633	88 / 400	13.42
Total (95% CI)	0.0801	0.0494	0.1272		

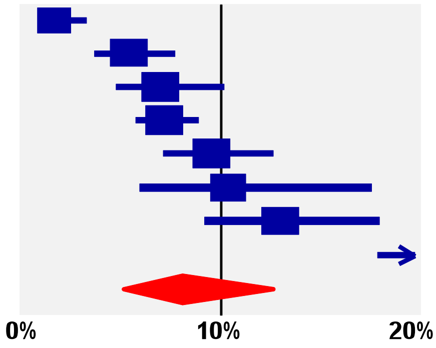

Supplement: File S1 — Forest plots for HIV, syphilis, gonorrhea, and chlamydia. Forest plots showing unadjusted estimates (squares) with 95% confidence intervals (bars) for HIV (Figure S1), syphilis (Figure S2), gonorrhea (Figure S3), and chlamydia (Figure S4) among male clients of female sex workers in China. The pooled prevalence estimate is represented as a red diamond. (PDF) [file pone.0071394.s003.pdf]
